# Isoechoic Renal Tumors: A Case Report and Literature Review

**DOI:** 10.3390/diagnostics16010014

**Published:** 2025-12-19

**Authors:** Nicola Sinatra, Giulio Geraci, Roberto Palumbo, Gaspare Oddo, Giuseppe Zichittella, Emanuele Cirafici, Alessandra Sorce, Giuseppe Mulè, Caterina Carollo

**Affiliations:** 1 Hemodialysis Unit, “Paolo Borsellino” Hospital, ASP Trapani, 91026 Marsala, Italy; sinatra.nicola@libero.it (N.S.); giuseppe.zichi@libero.it (G.Z.); 2 Faculty of Medicine and Surgery, Kore University, 94100 Enna, Italy; giulio.geraci@unikore.it; 3 Radiology Unit, “Paolo Borsellino” Hospital, ASP Trapani, 91026 Marsala, Italy; berto87vee@gmail.com; 4 Nephrology and Dialysis Unit, “Sant’Antonio Abate” Hospital, ASP Trapani, 91100 Trapani, Italy; direttore.nefrologiatp@asptrapani.it; 5 Department of Health Promotion Sciences, Maternal and Infant Care, Internal Medicine and Medical Specialties “G. D’Alessandro” (PROMISE), University of Palermo, 90127 Palermo, Italy; emanuele.cirafici@community.unipa.it (E.C.); alessandra.sorce@community.unipa.it (A.S.); giuseppe.mule@unipa.it (G.M.); 6 Unit of Nephrology and Hypertension, European Society of Hypertension Excellence Centre, University of Palermo, 90127 Palermo, Italy

**Keywords:** ultrasound, isoechoic renal tumor, CEUS

## Abstract

**Background and Clinical Significance**: Isoechoic renal tumors, defined as masses demonstrating echogenicity similar to normal renal parenchyma, represent a significant diagnostic challenge in contemporary ultrasonographic practice. These lesions, occurring in 5–12% of all renal masses, frequently escape detection on conventional ultrasound, leading to delayed diagnosis and potentially adverse oncological outcomes. Isoechoic renal tumors encompass both benign and malignant entities, with clear cell renal cell carcinoma representing 65–70% of malignant cases. Conventional ultrasound shows limited sensitivity (48–67%) for detecting isoechoic masses, while contrast-enhanced ultrasound achieves detection rates of 94–98%. Multiparametric MRI and dual-energy CT provide superior characterization, with accuracy rates of 85–92% for differentiating benign from malignant lesions. **Case Presentation:** We describe the case of an 80-year-old male in whom a 2.4 cm isoechoic renal mass was incidentally detected during abdominal ultrasound performed for chronic kidney disease monitoring. Contrast-enhanced CT confirmed a solid, hypervascular lesion with wash-out characteristics. Given the patient’s age, comorbidities, and tumor characteristics, multidisciplinary evaluation led to an active surveillance strategy. At 6-month follow-up, the lesion remained stable. **Conclusions:** Isoechoic renal tumors require multimodal diagnostic approaches and individualized management strategies. Emerging technologies, including artificial intelligence-enhanced ultrasound systems and radiomic-based decision support tools, are undergoing clinical validation and may improve detection and characterization. Investigational approaches such as liquid biopsy and novel PET tracers targeting carbonic anhydrase IX are in early development. Translation of these technologies into clinical practice will require prospective validation, standardization of protocols, and demonstration of cost-effectiveness.

## 1. Introduction

Renal tumors represent 2–3% of adult malignancies, with over 60% incidentally found during unrelated imaging [1]. Renal cell carcinoma (RCC) exhibits a significant global health burden, with approximately 431,000 new cases and 179,000 deaths annually worldwide [2]. Incidence rates vary considerably by geography, sex, and ethnicity, being highest in North America, Western Europe, and Australia/New Zealand [3,4]. Prognosis is strongly stage-dependent, with five-year survival ranging from 93% for localized disease to only 12% for metastatic RCC [1]. The social expenditure is considerable, with direct medical costs exceeding $5 billion annually in United States [5].

Among renal masses, isoechoic tumors offer unique diagnostic challenges due to their echogenicity matching normal renal parenchyma, potentially causing delayed diagnosis of malignant lesions [6].

The term “isoechoic” specifically refers to acoustic impedance properties resulting in similar sound wave reflection patterns compared to normal renal tissue [7]. Several factors contribute to this appearance, including tumor cellularity, vascularity, hemorrhage, necrosis, and cytoplasm-to-nucleus ratio within tumor cells [8]. Technical considerations are also crucial, as echogenicity assessment depends on ultrasound equipment settings and operator expertise, with studies showing that ultrasound findings may vary between different technical systems [9].

The prevalence of isoechoic renal tumors ranges from 5–10% when only perfectly isoechoic masses are operated on [10] to 30–40% when masses with echogenicity similar to normal parenchyma are included [11]. Small masses (<3 cm) are more likely to be isoechoic, with rates of 20–25% [12].

These masses include both benign and malignant entities such as Renal Cell Carcinoma (RCC), fat-poor angiomyolipoma, and oncocytoma [13]. Their sonographic similarity to normal tissue leads to frequent oversight during routine ultrasound, resulting in delayed treatment [10].

Conventional grayscale ultrasound demonstrates a sensitivity of 48–67% for detecting these lesions, with up to 30% of isoechoic masses missed on initial examination [14]. This detection challenge is clinically significant because isoechoic RCCs are typically larger at diagnosis than other echogenic variants, indicating prolonged undetected growth [15].

The aim of this article is twofold. First, we present a case report of an incidentally detected isoechoic renal mass in an elderly patient, illustrating the typical clinical scenario and diagnostic pathway. Second, we provide a comprehensive literature review examining the pathophysiology, diagnostic modalities, differential diagnosis, and current management strategies for isoechoic renal tumors, with particular emphasis on practical clinical recommendations aligned with current guidelines from the American Urological Association (AUA) and European Association of Urology (EAU).

## 2. Case Presentation

We report a case of incidentaloma of the kidney during an outpatient check-up. An 80-year-old man arrived at our Hemodialysis unit of the “P. Borsellino” Hospital in Marsala for a nephrological check-up. The patient had a medical history of chronic kidney disease (CKD) stage 3b under conservative treatment known for about 6 months, arterial hypertension on medication, no diabetes, chronic atrial fibrillation on oral anticoagulant drugs, benign prostatic hypertrophy and Alzheimer’s disease. His mean arterial pressure was 130/80 mmHG, HR 57 bpm. He referred no back pain, or constitutional symptoms such as weight loss or fatigue. Laboratory evaluation showed serum creatinine of 1.56 mg/dL (eGFR 41.3 mL/min/1.73 m^2^), hemoglobin 11.2 g/dL, and unremarkable urinalysis without microscopic hematuria.

**Figure 1:**
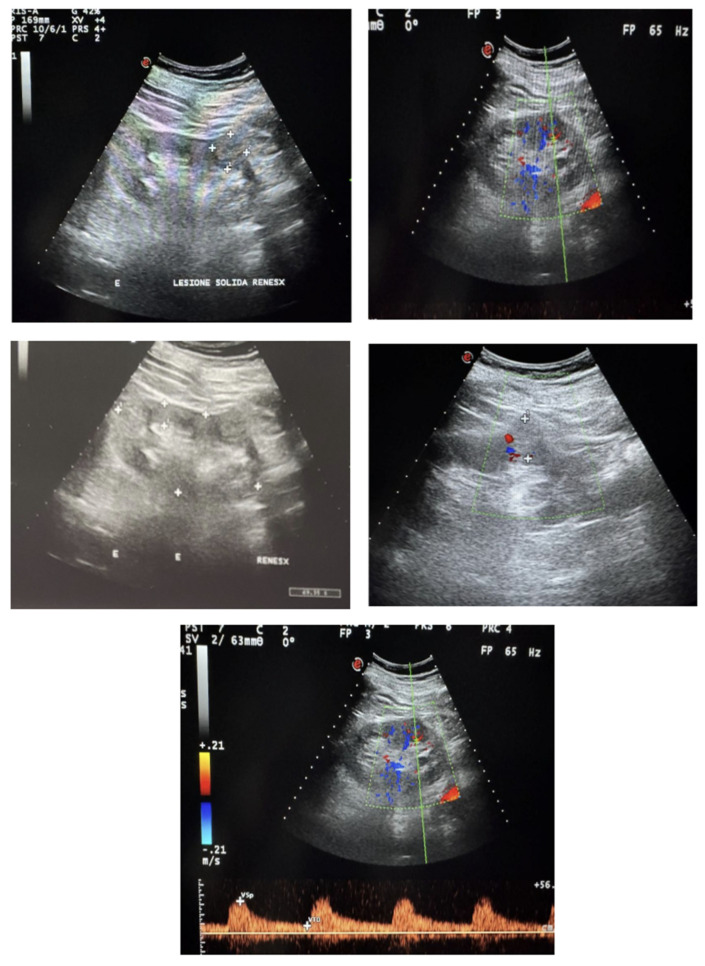
Left kidney with Isoechoic solid lesion at the lower middle third.

An ultrasound scanning was also carried out: it showed well-placed and normal sized kidneys, (Right longitudinal diameter was 10.26 cm, left longitudinal diameter was 10.92 cm). The parenchymal thickness was normal for the age, the echostructure was slightly hyperechoic with good cortico-medullary and parenchymopyelic differentiation. A posterior oblique longitudinal scan showed a solid isoechoic lesion at the lower middle third of the left kidney with a maximum diameter of 2.3 × 2.35 cm (Figure 1). On color-Doppler integration, an alteration of the vascular architecture was evident.

**Figure 2:**
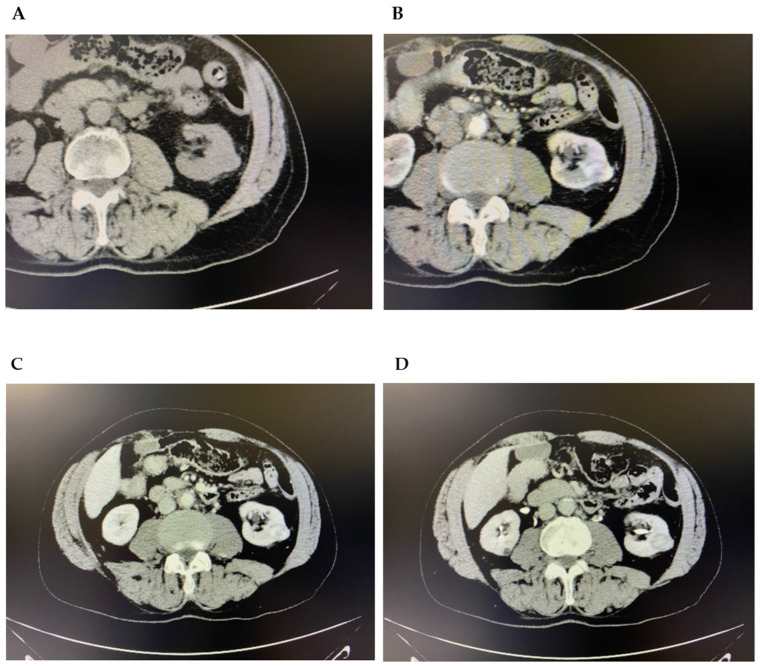
CT scan of the abdomen complete, at baseline (**A**) and after MDC arterial phase (**B**), portal phase (**C**) and late phase (**D**).

On the basis of ultrasound evidence, due to the absence of Contrast-Enhanced Ultrasound (CEUS) in our unit, the patient underwent 2nd-level investigations. An abdominal CT scan, before and after contrast-medium administration, confirmed the renal clinical picture by reporting a partially exophytic cortical nodulation in the middle third of the posterior margin of the left kidney of the DT max of about 2.4 cm, nuancedly hypodense. The lesion also showed intense post-contrast enhancement in the arterial and portal phase of the study, followed by a clear wash-out in the late phase. Signs of neoangiogenesis were also detected in the immediately contiguous perirenal fat. No continuity with the nearest calyceal groups was present (Figure 2).

**Differential Diagnosis Considerations**

Based on the imaging characteristics, the differential diagnosis included clear cell RCC (most likely due to the enhancement pattern), papillary RCC (less likely due to hypervascularity), chromophobe RCC, oncocytoma, and fat-poor angiomyolipoma.

**Clinical Management and Follow-up**

The patient was referred for urological and oncological evaluation. Given his age (80 years), comorbidities (CKD stage 3b, Alzheimer’s disease), and tumor characteristics (T1a, 2.4 cm, favorable location with RENAL nephrometry score of 5), the multidisciplinary tumor board discussed management options including active surveillance, thermal ablation, and partial nephrectomy.

After shared decision-making with the patient and family, considering his life expectancy (estimated 5–7 years based on Charlson Comorbidity Index of 5) and the slow growth rate typical of small isoechoic masses (0.28 cm/year on average), an initial active surveillance approach was chosen. The surveillance protocol included abdominal MRI at 3 months, then every 6 months for 2 years, with surgical intervention planned if growth exceeds 0.5 cm/year or if the tumor reaches 4 cm.

At 6-month follow-up MRI, the lesion remained stable at 2.4 cm with unchanged imaging characteristics, supporting the appropriateness of continued surveillance in this elderly patient with significant comorbidities.

**Case Discussion**

This case exemplifies several key challenges addressed in this review. First, the isoechoic nature of the lesion made detection on conventional ultrasound difficult, with identification relying on subtle contour changes and Doppler abnormalities rather than echogenic contrast. Second, the incidental discovery during routine CKD monitoring reflects the predominant presentation pattern of isoechoic masses (75–80% incidentally detected). Third, the diagnostic pathway illustrates the necessity of multimodal imaging when conventional ultrasound findings are indeterminate. Finally, the management decision demonstrates the importance of individualized approaches incorporating patient factors, tumor characteristics, and shared decision-making, particularly in elderly patients where competing mortality risks may exceed cancer-specific risks.

**Limitations of the Case**

Several limitations of this case should be underlined. Most notably, the absence of histopathological confirmation makes the diagnosis presumptive, based solely on imaging characteristics. While the enhancement pattern—with intense arterial enhancement followed by clear wash-out—is highly suggestive of clear cell renal cell carcinoma, it is not possible to definitively exclude other entities such as oncocytoma or fat-poor angiomyolipoma. The decision to avoid biopsy in this case was based on the multidisciplinary assessment that the procedural risks and potential impact on management were outweighed by the patient’s advanced age, cognitive impairment limiting cooperation, and the selected surveillance strategy.

Additionally, the relatively short follow-up period of 6 months limits conclusions regarding long-term tumor behavior and the appropriateness of continued surveillance. Finally, the unavailability of contrast-enhanced ultrasound (CEUS) at our institution precluded its use as an intermediate diagnostic step, which might have provided additional characterization without radiation exposure.

## 3. Epidemiology and Natural History of Isoechoic Renal Tumors

### 3.1. Epidemiology

The true prevalence of isoechoic renal tumors is not established, as many lesions are identified only through cross-sectional imaging [16]. Available data suggest isoechoic appearance accounts for approximately 5–12% of all RCC, though this likely underestimates true prevalence due to detection bias [17]. In a large retrospective analysis of surgically resected renal masses, Kim and colleagues found approximately 8–9% were classified as isoechoic on preoperative ultrasound [17].

The propensity for isoechoic appearance varies significantly among histological subtypes, reflecting underlying differences in tumor architecture and composition [18,19,20,21]. Table 1 summarizes the prevalence and isoechoic rates of common renal tumor subtypes.

### 3.2. Geographic and Demographic Patterns and Risk Factors

Isoechoic renal tumors show male predominance with a male-to-female ratio of approximately 1.5:1, less pronounced than the 2:1 ratio for all RCCs [22]. Mean age at diagnosis is 62–65 years, slightly older than the overall RCC population, possibly reflecting diagnostic delays [23]. Geographical variations exist, with higher detection rates in countries with widespread ultrasound screening programs like Japan and South Korea [24].

Racial and ethnic disparities documented for RCC generally also apply to isoechoic tumors. African American patients demonstrate 20% higher incidence rates and 30% higher mortality compared to Caucasian patients, attributed to a combination of biological factors, socioeconomic determinants, and healthcare access disparities [4]. Hispanic and Asian American populations show lower overall incidence but similar stage-adjusted survival [25].

Risk factors overlap with general RCC risk factors, including smoking, obesity, hypertension, and chronic kidney disease [26]. Patients with von Hippel–Lindau disease frequently develop small isoechoic lesions difficult to distinguish from cysts [27]. Genetic syndromes like Birt–Hogg–Dubé syndrome are favoring conditions for hybrid oncocytic tumors that frequently appear isoechoic [28].

### 3.3. Natural History and Clinical Presentation

Longitudinal studies suggest that isoechoic RCCs seem to be larger at diagnosis, with mean sizes of 4.5–5.0 cm compared to 3.5–4.0 cm for all RCCs, thus postulating periods of undetected growth [29]. Growth rates have an average value of 0.3–0.4 cm annually for small isoechoic RCCs, though high-grade subtypes may exceed 0.5 cm per year [30]. These growth patterns underscore the importance of active surveillance protocols for small renal masses, as early detection and monitoring can prevent progression to advanced stages while avoiding overtreatment in indolent cases.

Transition from isoechoic to heteroechoic appearance often correlates with growth beyond 4 cm or hemorrhage onset or cystic degeneration [11].

Clinical presentation shows even higher rates of incidental detection than typical renal masses, approaching 75–80% in contemporary series [31]. When symptomatic, these tumors typically present at more advanced stages, with the classic triad of flank pain, hematuria, and palpable mass occurring in 15–20% of patients compared to less than 10% for all RCCs [32]. Detection patterns evolved with imaging advances, particularly contrast-enhanced ultrasound, which can identify up to 95% of isoechoic masses missed by conventional ultrasound [33].

## 4. Pathophysiology of Isoechoic Appearance

The isoechoic appearance of renal tumors on ultrasound reflects specific pathophysiological characteristics that result in their acoustic properties. At molecular level, tissue echogenicity depends on interfaces between structures with different acoustic impedances, primarily determined by cellular density, nuclear-to-cytoplasmic ratio, and extracellular matrix composition [34]. Isoechoic renal tumors typically show cellular architecture that closely mimics normal renal tissue, with uniform cell distribution and minimal stromal reaction, resulting in homogeneous sound wave reflection patterns [35].

Tumor cellularity directly influences echogenicity. Quantitative studies demonstrated that isoechoic RCCs typically exhibit cell densities of 60–80% of tumor volume, comparable to normal renal parenchyma [36].

Secondary changes including hemorrhage, necrosis, calcification, and cystic degeneration alter tumor echogenicity, typically creating hypoechoic or hyperechoic areas that make detection easier [37]. Histopathological analysis shows that isoechoic tumors have significantly lower rates of these secondary changes, with less than 10% showing hemorrhage or necrosis compared to 40–50% in heteroechoic tumors [8]. The absence of these features maintains the acoustic similarity to surrounding renal parenchyma, explaining why larger isoechoic tumors may remain undetected while smaller heteroechoic lesions are readily identified [38].

In our patient, the isoechoic appearance on initial ultrasound is consistent with these pathophysiological principles. The lesion’s homogeneous echotexture, similar to surrounding renal parenchyma, suggested a uniform cellular architecture and absence of hemorrhage or necrosis—features subsequently confirmed on contrast-enhanced CT, which demonstrated a solid, homogeneously enhancing mass.

## 5. Imaging Modalities and Diagnostic Challenges

The detection and characterization of isoechoic renal tumors requires a multimodal imaging approach. This section reviews the capabilities, limitations, and clinical applications of available imaging technologies, emphasizing practical guidance for clinical decision-making. Van Oostenbrugge et al. [39] provided a comprehensive pictorial review of diagnostic imaging for solid renal tumors, emphasizing that accurate characterization requires integration of multiple imaging modalities, each contributing unique information about tumor morphology, vascularity, and tissue composition. Their work highlights how systematic evaluation across imaging platforms improves diagnostic confidence, particularly for lesions with ambiguous features on single-modality assessment.

### 5.1. Ultrasound Evaluation

Conventional grayscale ultrasound is the primary screening modality for renal masses, although its sensitivity for detecting isoechoic tumors is significantly limited, ranging from 48 to 67% if compared to over 85% for all renal masses [40]. The fundamental challenge lies in the lack of echogenic contrast between tumor and normal parenchyma, with detection often relying on secondary signs such as contour alterations, loss of corticomedullary differentiation, or displacement of normal structures [41]. Technical factors including transducer frequency, gain settings, and patient body habitus further impact detection rates, with obesity reducing sensitivity to as low as 40% for isoechoic masses [42].

Color and power Doppler ultrasound provide additional information about tumor vascularity but show limited utility for isoechoic lesions. Isoechoic RCCs frequently exhibit peripheral and central vascularity patterns that are not distinguishable from normal renal parenchyma, with resistive indices overlapping significantly with benign lesions [43]. Spectral Doppler analysis may reveal subtle differences in peak systolic velocities, though these findings lack sufficient specificity for reliable characterization [44].

Contrast-enhanced ultrasound achieves sensitivity rates of 94–98% for detecting isoechoic tumors previously missed on conventional ultrasound [15] thus limiting radiation exposure, particularly valuable for patients requiring serial imaging or those with contraindications to iodinated contrast. Malignant lesions typically demonstrate heterogeneous enhancement, early washout, and perilesional rim enhancement, features that guide clinical decision-making regarding the need for further investigation or intervention [45]. Quantitative CEUS parameters, including time-intensity curves and perfusion indices, show promise for differentiating benign from malignant isoechoic masses, with accuracy rates approaching 85–90% [46]. A meta-analysis demonstrated that CEUS achieved 88% sensitivity and 80% specificity for differentiating malignant from benign isoechoic masses, with diagnostic performance comparable to contrast-enhanced MRI but at substantially lower cost [47]. CEUS-guided percutaneous biopsy improves diagnostic yield from 76% to 94%, directly impacting treatment planning by providing accurate histological diagnosis in challenging cases [48]. Granata et al. [49,50] extensively demonstrated that CEUS significantly improves the detection and characterization of isoechoic renal masses through real-time visualization of tumor vascularity, identifying up to 95% of isoechoic lesions missed on conventional B-mode ultrasound.

### 5.2. Elastography

Ultrasound elastography has emerged as a valuable complementary technique for isoechoic renal tumors by assessing tissue stiffness properties. Shear wave elastography (SWE) provides quantitative stiffness measurements, with malignant isoechoic masses demonstrating significantly higher mean elasticity values (28.7 ± 12.3 kPa) compared to benign lesions (15.2 ± 6.8 kPa) and normal renal cortex (7.5 ± 2.1 kPa), achieving diagnostic accuracy of 84% for differentiating malignant from benign isoechoic masses [51]. Strain elastography, which evaluates tissue deformation under compression, reveals that isoechoic RCC exhibit strain ratios >2.5 in 86% of cases, with clear cell RCC showing higher stiffness values than papillary or chromophobe subtypes [52].

Elastography and conventional B-mode ultrasound together improve sensitivity for detecting isoechoic masses from 67% to 82%, particularly for lesions 2–4 cm that remain challenging [53].

Limitations exist including depth dependency, with accuracy decreasing for masses >8 cm from the skin surface, and overlap in stiffness values between oncocytomas and well-differentiated RCC, necessitating integration with other imaging parameters for optimal characterization [54].

### 5.3. Computed Tomography

Multidetector computed tomography (MDCT) with multiphasic contrast enhancement represents the gold standard for renal mass evaluation. For isoechoic tumors, the combination of unenhanced, corticomedullary, nephrographic, and excretory phases achieves detection rates exceeding 95% [55]. During the nephrographic phase, isoechoic masses typically enhance less than normal parenchyma, creating a contrast difference absent on ultrasound [56].

Dual-energy CT (DECT) offers advanced characterization capabilities through material decomposition and virtual monoenergetic imaging. DECT improves detection of isoechoic masses by 15–20% compared to conventional CT, particularly for lesions smaller than 3 cm [57]. Iodine quantification maps enable precise assessment of enhancement patterns, with malignant isoechoic tumors demonstrating iodine concentrations of 2.5–4.0 mg/mL compared to 1.0–2.0 mg/mL in benign lesions [58]. Virtual unenhanced images reduce radiation exposure by eliminating the need for true unenhanced acquisitions while maintaining diagnostic accuracy [59].

### 5.4. Magnetic Resonance Imaging

MRI provides superior soft tissue contrast for evaluating isoechoic renal masses, with multiparametric protocols achieving sensitivity and specificity exceeding 90% for malignancy detection [60]. T2-weighted imaging reveals internal architecture, with isoechoic RCCs typically showing intermediate to high signal intensity, while chemical shift imaging detects microscopic fat in clear cell RCCs, appearing as signal dropout on opposed-phase images [61]. Dynamic contrast-enhanced MRI with gadolinium demonstrates enhancement patterns similar to CT but with superior temporal resolution, enabling detailed assessment of perfusion kinetics [62].

Diffusion-weighted imaging (DWI) adds functional information about tissue cellularity and membrane integrity. Isoechoic malignant tumors typically show restricted diffusion with apparent diffusion coefficient (ADC) values of 1.0–1.5 × 10^−3^ mm^2^/s, significantly lower than benign lesions [63]. The combination of DWI with conventional sequences improves characterization accuracy to 85–92% for indeterminate isoechoic masses [64]. Advanced techniques including intravoxel incoherent motion (IVIM) and diffusion kurtosis imaging show promise for further refinement of tissue characterization [65].

### 5.5. Diagnostic Challenges and Pitfalls

Several factors complicate the imaging evaluation of isoechoic renal tumors. Small lesions remain difficult, with detection rates dropping below 70% for isoechoic masses under 2 cm even with advanced imaging [11]. Partial volume averaging effects at tumor margins can obscure small isoechoic lesions, particularly those located in the renal poles or adjacent to the collecting system [66]. Patient factors including respiratory motion, cardiac pulsation, and inability to suspend respiration degrade image quality and reduce diagnostic confidence [67].

Mimics of isoechoic tumors create diagnostic dilemmas. Prominent columns of Bertin, focal renal hypertrophy, and inflammatory pseudotumors may simulate isoechoic masses on imaging [68]. Differentiating fat-poor angiomyolipomas from RCC remains particularly challenging, as both can appear isoechoic with similar enhancement patterns [69]. Integration of multiple imaging modalities and clinical correlation improves diagnostic accuracy, though histological confirmation through biopsy or surgical resection often remains necessary for definitive diagnosis and appropriate treatment planning [70].

## 6. Differential Diagnosis and Clinical Decision-Making

The differential diagnosis of isoechoic renal masses encompasses a broad spectrum of benign and malignant entities. RCC variants constitute the primary concern, with clear cell RCC representing 65–70% of isoechoic malignant tumors, followed by papillary (15–20%) and chromophobe (5–10%) subtypes [71]. On advanced imaging, clear cell RCC demonstrates avid arterial enhancement (>100 HU) compared to moderate enhancement (40–60 HU) in papillary RCC, though considerable overlap exists for isoechoic presentations, limiting the reliability of enhancement patterns alone for subtype determination [72].

Among benign lesions, oncocytoma accounts for 3–7% of all renal tumors and presents as isoechoic in approximately 40% of cases [73]. The classic central stellate scar, when present, may suggest this diagnosis; however, this finding appears in only 30–35% of oncocytomas and remains difficult to identify in isoechoic lesions on conventional imaging [74]. Fat-poor angiomyolipoma occurs in 5% of angiomyolipomas and appears isoechoic in 60–70% of cases, with CT attenuation values overlapping significantly with RCC (30–45 HU), often necessitating biopsy for definitive characterization [69,75].

Complex cysts and infectious processes further complicate differential diagnosis. Hemorrhagic or proteinaceous cysts may appear isoechoic on ultrasound, with CT demonstrating high attenuation (50–70 HU) on unenhanced images without enhancement, a finding that reliably excludes solid neoplasm [32]. Focal pyelonephritis and renal abscesses can present as isoechoic masses, particularly in early stages before liquefaction occurs; clinical correlation and short-interval follow-up imaging are essential for differentiation, avoiding unnecessary intervention [76].

### 6.1. Clinical Decision-Making Framework

The management of isoechoic renal masses requires systematic evaluation integrating clinical factors, imaging characteristics, and patient factors, following established clinical guidelines. According to current EAU and AUA recommendations [77,78], initial assessment focuses on determining enhancement presence and degree, with lesions demonstrating >15–20 HU enhancement requiring further evaluation as presumed solid tumors [79]. For confirmed solid isoechoic masses, size remains the primary determinant of management strategy, with masses <4 cm (T1a) considered suitable for active surveillance in 30–40% of appropriately selected patients [80].

**Figure 3:**
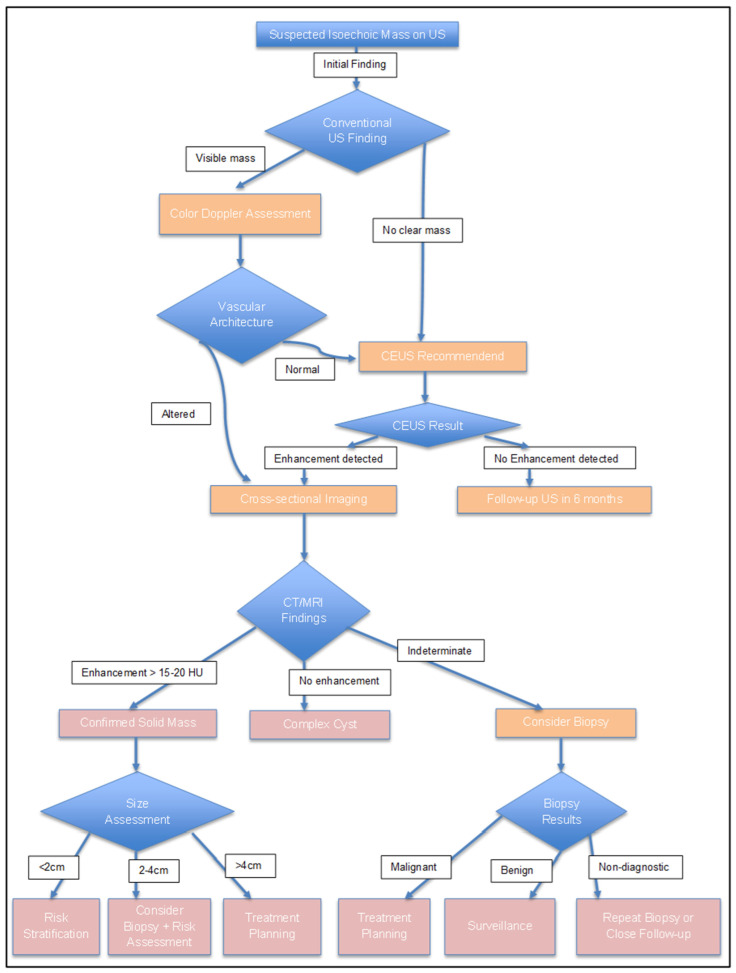
Diagnostic flow-chart for Isoechoic Renal Masses.

Patient factors significantly influence management decisions. Age and comorbidities guide the choice between intervention and surveillance, with competing mortality risk exceeding cancer-specific mortality for patients >75 years with masses <3 cm [81]. Renal function assessment is important, as 26–30% of patients with isoechoic RCC have chronic kidney disease at presentation, influencing the choice between radical and partial nephrectomy [82]. Life expectancy calculations using validated tools demonstrate that patients with <5 years expected survival rarely benefit from intervention for asymptomatic isoechoic masses <4 cm [83] (Figure 3).

### 6.2. Role of Renal Mass Biopsy

Percutaneous renal mass biopsy is a valuable tool for characterizing indeterminate isoechoic lesions, with diagnostic rates of 85–92% and complication rates <5% in experienced centers [84]. For isoechoic masses, different studies show management changes in 40–45% of cases following biopsy [85]. Core biopsy demonstrates superior diagnostic yield compared to fine-needle aspiration, providing adequate tissue for histological subtyping in 87% of cases and grade assessment in 76% [86].

However, clinicians should be aware of limitations and relative contraindications. Non-diagnostic biopsy rates range from 8 to 15%, and sampling error may occur in heterogeneous tumors, potentially underestimating tumor grade [84,85]. Relative contraindications include unfavorable anatomical location (central or hilar masses near major vessels), uncorrectable coagulopathy, patient inability to cooperate with breath-holding instructions, and lesions with high suspicion for urothelial carcinoma where tract seeding represents a concern [87,88]. In these situations, direct surgical excision or alternative imaging strategies may be preferred [89].

Immunohistochemistry enhances diagnostic accuracy for isoechoic masses, with panels including PAX8, carbonic anhydrase IX, and CK7 achieving 90–95% accuracy for RCC subtype determination [90]. Molecular markers enhance traditional histology: SDHB and fumarate hydratase (FH) immunohistochemistry identify SDH-deficient and FH-deficient RCC, respectively [91], while CK7, CD117, and vimentin expression patterns help differentiate chromophobe RCC from clear cell RCC [92].

Genomic classifiers distinguishing oncocytoma from chromophobe RCC with 85% accuracy when morphology alone results to be insufficient [93].

### 6.3. Treatment Selection Algorithms

Contemporary management algorithms for isoechoic renal masses emphasize risk-adapted strategies based on tumor and patient characteristics. For masses <4 cm in favorable locations (RENAL score ≤6), partial nephrectomy remains a standard procedure, achieving oncologic outcomes equivalent to radical nephrectomy with 5-year cancer-specific survival of 95–97% [94]. Thermal ablation provides alternative treatment for selected isoechoic masses <3 cm, particularly in patients with comorbidities precluding surgery, achieving local control rates of 88–94% [95].

Active surveillance protocols specifically designed for isoechoic masses incorporate imaging every 3–6 months initially, with intervention triggered by growth >0.5 cm/year or size >4 cm [96]. Decision analysis models demonstrate that surveillance strategies optimize quality-adjusted life years for elderly patients with competing risks, while immediate intervention benefits younger patients with longer life expectancy [97].

## 7. Current Treatment Strategies and Therapeutic Approaches

### 7.1. Surgical Management

Partial nephrectomy remains the gold standard treatment for localized isoechoic renal masses ≤7 cm, achieving oncologic outcomes comparable to radical nephrectomy while preserving renal function [98]. Intraoperative ultrasound significantly improves tumor identification for isoechoic lesions [99], with contemporary series reporting high negative margin rates and low local recurrence rates at 5 years [100].

Robotic-assisted approaches offer advantages for complex cases through enhanced visualization, including indocyanine green fluorescence imaging for margin delineation [101,102,103]. Radical nephrectomy remains indicated for larger masses (>7 cm), unfavorable anatomic configurations, or locally advanced tumors with venous involvement [104,105].

### 7.2. Ablative Therapies

Thermal ablation offers effective treatment for selected isoechoic masses ≤3 cm, particularly in patients with comorbidities precluding surgery or requiring nephron preservation. Among ablative modalities, cryoablation demonstrates the highest complete response rates with favorable long-term local control [95,106]. Radiofrequency ablation achieves good complete response rates, though technical challenges may arise from similar tissue impedance between tumor and parenchyma [107,108]. Microwave ablation offers faster treatment times with promising early results, though long-term data remain limited [109]. Contrast-enhanced ultrasound guidance improves targeting accuracy across all ablative modalities [110].

### 7.3. Active Surveillance Protocols

Active surveillance is appropriate for small isoechoic masses in selected patients, with prospective registries demonstrating low metastatic progression rates for masses ≤4 cm over 5 years [96]. Standardized protocols employ cross-sectional imaging every 3–6 months initially, then annually, with intervention triggered by significant growth or symptom development [96,111]. Patient selection criteria emphasize advanced age, significant comorbidities, and small tumor size [80,112].

### 7.4. Systemic Therapy for Advanced Disease

For metastatic isoechoic RCC, contemporary systemic regimens have significantly improved outcomes. Combination immunotherapy with checkpoint inhibitors achieves meaningful response rates in treatment-naïve patients with intermediate/poor-risk disease [113]. Tyrosine kinase inhibitor combinations demonstrate efficacy for clear cell histology, with favorable progression-free survival [114]. Treatment selection should be guided by IMDC risk stratification, histologic subtype, and patient performance status. The isoechoic appearance of the primary tumor does not independently influence systemic therapy response; however, histologic subtype—confirmed through biopsy or surgical specimen—remains critical for treatment planning.

Table 2 provides a concise clinical summary of the main diagnostic and therapeutic considerations for isoechoic renal tumors.

## 8. Future Perspectives and Emerging Technologies

**Technologies in Advanced Clinical Validation**

Artificial intelligence-enhanced ultrasound systems and radiomic-based decision support tools represent the most mature emerging technologies for renal mass evaluation. These approaches leverage existing imaging infrastructure, requiring software integration rather than new hardware acquisition [115,116]. Studies have demonstrated promising diagnostic performance for differentiating benign from malignant lesions, including angiomyolipoma without visible fat distinguished from RCC [117,118]. However, standardization of imaging protocols and feature extraction methods remains a critical barrier [115,118]. Among emerging technologies discussed, AI-enhanced imaging and radiomics are closest to potential clinical implementation, though prospective studies demonstrating impact on clinical decision-making and patient outcomes are needed before widespread adoption can be recommended.

**Technologies in Early Clinical Development**

Liquid biopsy technologies, including circulating tumor DNA and circulating tumor cell analysis, remain promising investigational tools for renal masses. Current evidence demonstrates feasibility for detection in RCC patients; however, clinical utility for primary tumor characterization—as opposed to metastatic disease monitoring—has not been established [119]. Prospective validation demonstrating added value beyond imaging-based approaches is required.

Novel PET tracers targeting carbonic anhydrase IX, particularly ^89^Zr-girentuximab, have demonstrated feasibility for lesion detection in metastatic clear cell RCC [120]. Further studies are needed to evaluate potential applications in primary tumor characterization.

**Technologies in Preclinical or Early Experimental Phases**

Photoacoustic imaging remains in preclinical and early human studies [121,122]. Significant technical hurdles persist, including dedicated equipment development and regulatory pathway establishment. This technology is furthest from clinical implementation among those discussed.

**Cost-Effectiveness and Clinical Impact**

Health economic evaluation represents a critical evidence gap for all emerging technologies in renal mass characterization. Demonstration of technical accuracy is insufficient; technologies must ultimately prove beneficial for therapeutic decision-making and patient outcomes. Current evidence for most technologies addresses primarily technical feasibility and diagnostic accuracy, with limited data regarding impact on clinical management.

Cost-effectiveness will vary across healthcare settings. Software-based solutions leveraging existing equipment face lower implementation barriers than technologies requiring new infrastructure.

**Future Directions**

Prospective multicenter validation with standardized protocols, comparative effectiveness studies, and health economic analyses represent research priorities. Collaborative efforts between academic centers, industry, and regulatory agencies will be essential to translate promising technologies into practice.

## 9. Conclusions

Isoechoic renal tumors pose a significant diagnostic challenge due to their acoustic similarity to normal renal parenchyma.

We showed the typical clinical scenario and underlined the significance of individualized approaches that account for patient age, comorbidities, and tumor characteristics.

From a diagnostic perspective, conventional ultrasound has limited sensitivity for isoechoic lesions, and detection often relies on indirect signs. Contrast-enhanced ultrasound significantly improves detection rates, while multiphasic CT and multiparametric MRI provide definitive characterization. A multimodal approach is recommended when initial ultrasound findings are indeterminate.

From a therapeutic perspective, management should be risk-adapted. Active surveillance is appropriate for selected patients with small masses and significant competing mortality risks. Partial nephrectomy remains the standard treatment for localized disease in surgical candidates, while thermal ablation offers an alternative for patients with contraindications to surgery.

Emerging technologies, including artificial intelligence-enhanced imaging and radiomic analysis, may improve future detection and characterization, though clinical validation is ongoing.

Clinicians should maintain a high index of suspicion for isoechoic masses during renal ultrasound and pursue further imaging when indirect signs suggest an underlying lesion. Multidisciplinary evaluation ensures optimal individualized management.

## Figures and Tables

**Table 1:** Histological Subtypes of Isoechoic Renal Masses.

Histological Subtype	Overall Prevalence (% of Renal Tumors)	Isoechoic Rate (%)	Key Histological Features
Clear cell RCC	70–75% of RCCs	6–10%	Abundant clear cytoplasm, compact alveolar pattern, delicate vascular network
Papillary RCC	10–15% of RCCs	15–20%	Small cuboidal cells, papillary/tubular patterns, minimal hemosiderin
Chromophobe RCC	5% of RCCs	25–30%	Large polygonal cells, perinuclear halos, numerous mitochondria
Oncocytoma	3–7% of renal tumors	35–40%	Densely eosinophilic cytoplasm, nested architecture, central scar (30%)
Fat-poor AML	5% of AMLs	60–70%	Minimal adipose tissue, smooth muscle predominant, thick-walled vessels

**Table 2:** Summary table: Isoechoic Renal Tumors.

Category	Key Findings
**Definition**	Renal masses with echogenicity equal to or similar to normal renal parenchyma on ultrasound
**Diagnostic Approach**	Conventional US limited (sensitivity < 70%); CEUS and multiphasic CT/MRI recommended for characterization
**Benign vs. Malignant**	Fat-poor AML and oncocytoma frequently appear isoechoic; biopsy recommended when diagnosis uncertain
**Treatment Selection**	Partial nephrectomy (masses ≤ 7 cm, favorable anatomy); Ablation (≤3 cm, comorbid patients); Active surveillance (small masses, elderly, significant comorbidities)
**Clinical Challenges**	Delayed detection due to sonographic similarity to parenchyma; differentiation from benign lesions; management in patients with CKD

## Data Availability

The data presented in this study are available on request from the corresponding author.
